# Ovarian Dermoid Tumor

**DOI:** 10.7759/cureus.27233

**Published:** 2022-07-25

**Authors:** Mya St. Louis, Rohan Mangal, Thor S Stead, Marcos Sosa, Latha Ganti

**Affiliations:** 1 Biology, Florida Virtual School, Sebring, USA; 2 Medicine, Johns Hopkins University, Baltimore, USA; 3 Medicine, University of Miami Miller School of Medicine, Miami, USA; 4 Medicine, Warren Alpert Medical School of Brown University, Providence, USA; 5 Obstetrics and Gynecology, Lakeland Regional Health, Lakeland, USA; 6 Emergency Medicine, HCA Florida Ocala Hospital, Ocala, USA; 7 Emergency Medicine, Envision Physician Services, Plantation, USA; 8 Emergency Medicine, University of Central Florida College of Medicine, Orlando, USA

**Keywords:** emergency medicine, cyst, tumor, ovary, ovarian dermoid tumor

## Abstract

Ovarian dermoid cysts are a common benign tumor. Although there is often a genetic component to this abnormality, this report discusses the case of a patient with an ovarian dermoid tumor with no family history of gynecological cancer. The diagnosis, surgical management, and malignancy of ovarian dermoid cysts are discussed.

## Introduction

Ovarian dermoid cysts, or mature cystic teratomas (MCT), are the most common benign ovarian tumor in adults and adolescents. They make up 70% of benign masses before menopause and 20% post-menopause [1,2]. They are characterized by adult ectodermal, mesodermal, and endodermal tissue and can contain skin, hair, teeth, fat and muscle, and even thyroid and brain tissue [1,3]. When an MCT contains primarily ectodermal tissue, it is described as a dermoid cyst [3].

One pathophysiological hypothesis about their origins is that they develop from the self-fertilization of an oocyte [4]. There are also male reproductive tumors. The compositional difference between an ovarian tumor and the testicular teratoma, the male gonadal tumor, is that ovarian tumors are typically cystic, while testicular tumors are typically solid [5]. A study has shown that patients with a first-degree relative with an ovarian dermoid cyst were at a greater risk of developing one themselves, so the cause is likely in part genetic [4]. The authors present the case of a premenopausal woman with no history of ovarian cancer, in whom imaging revealed a dermoid tumor.

## Case presentation

A 38-year-old woman was transported to the emergency department (ED) experiencing left-sided flank pain and bloating. She had mild discomfort the night before, but it did not cause any disturbances in sleep. However, early the next morning, she had a sudden onset of pain described as radiating from her lower left quadrant to her midline. The patient had a history of polycystic ovarian disease and had not menstruated in the past few months. Her medical history was also significant for hypothyroidism and hypertension. After a COVID-19 infection, she had noticed hypoglycemic episodes and near-fainting spells that have caused her to take time off of work. She had no known history of diabetes, breast, or ovarian cancer. She had never smoked. Her past surgical history included a Cesarean section and tonsillectomy. Her vital signs included normal pulse oximetry of 98% and pulse of 91 beats per minute. She had a temperature of 97.8ºF and a respiratory rate of 20 breaths per minute. Her blood pressure was 165/101 mmHg. Physical examination was unremarkable except for guarding and tenderness in the left lower quadrant. Laboratory analyses were unremarkable except for a mild leukocytosis. The patient also had elevated immature granulocytes, neutrophils, lymphocytes, and monocytes (Table 1). 

**Table 1:** Hematology and chemistry profiles of the patient

	Normal range	Patient results
Hematology		
White blood cells	4.1 - 9.3 K/mm^3^	13.4 K/mm^3^
Red blood cells	3.28 - 5.50 M/mm^3^	4.89 M/mm^3^
Hemoglobin	12.1 - 15.1 gm/dL	13.1 gm/dL
Hematocrit	35.5 - 46.9%	40.40%
Platelets	150 - 450 K/mm^3^	298 K/mm^3^
Absolute basophils	0.0 - 0.2 K/mm^3^	0.1 K/mm^3^
Nucleated red blood cells %	0 - 0%	0%
Immature granulocytes	0 - 0 K/mm^3^	0.1 K/mm^3^
Neutrophils	1.4 - 6.5 K/mm^3^	8.3 K/mm3
Lymphocytes	1.2 - 3.4 K/mm^3^	4.2 K/mm3
Monocytes	0.1 - 0.6 K/mm^3^	0.7 K/mm^3^
Eosinophils	0 - 0.7 K/mm^3^	0.2 K/mm^3^
Chemistry		
Sodium	135 - 145 mmol/L	136 mmol/L
Potassium	3.5 - 5.3 mmol/L	4.3 mmol/L
Chloride	98 - 107 mmol/L	103 mmol/L
Carbon dioxide	21 - 32 mmol/L	23 mmol/L
Anion gap	8 - 12 mEq/L	10 mEq/L
Blood urea nitrogen	7 - 18 mg/dL	9 mg/dL
Creatinine	0.6 - 1.3 mg/dL	0.6 mg/dL
Glucose	74 - 106 mg/dL	142 mg/dL
Calcium	8.4 - 10.2 mg/dL	10.2 mg/dL

The patient had a small amount of blood in the urine, large leukocyte esterase, and elevated white blood cells (WBCs) in the urine (Table 2). 

**Table 2:** Urinalysis of the patient WBC - white blood cells; RBC - red blood cells; HCG - chorionic gonadotropin; Hpf - high power field

Urinalysis	Normal range	Patient result
Urine color	Yellow	Yellow
Urine appearance	Clear	Clear
Urine pH	5.0 - 8.5	6.0
Urine specific gravity	1.005 to 1.030	1.015
Urine protein	Negative	Negative
Urine glucose (stick; mg/dL)	Negative	Negative
Urine ketones	Negative	Negative
Urine blood	Negative	Small
Urine nitrate	Negative	Negative
Urine bilirubin	Negative	Negative
Urine urobilinogen	0.2 - 1.0 EU/dL	0.2 EU/dL
Urine leukocyte esterase	Negative	Large
Urine RBC	0 - 5 /Hpf	0-5 /Hpf
Urine WBC	0 - 5 /Hpf	10-20 /Hpf
Urine WBC clumps	Negative	Rare
Urine epithelial cells	0 - 5 /Hpf	0-5
Urine HCG, qual	Negative	Negative

**Figure 1:**
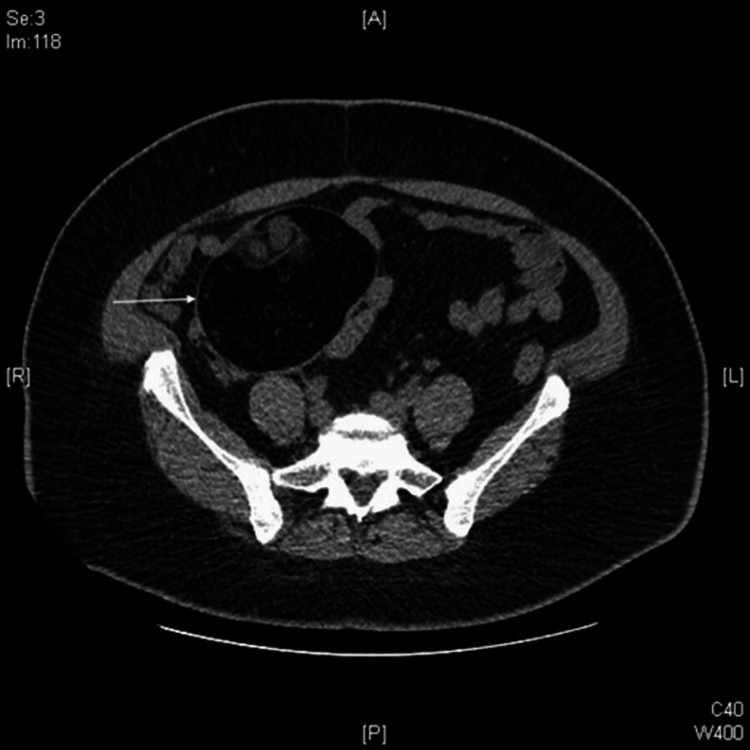
Abdominal and pelvic CT Arrow denotes ovarian mass.

Noncontrast computed tomography (CT) of the abdomen and pelvis showed a large right adnexal mass made up of solid and fatty tissue. Differential diagnoses included a large teratoma that could potentially be malignant (Figure 1), prompting ultrasonographic evaluation. 

**Figure 2:**
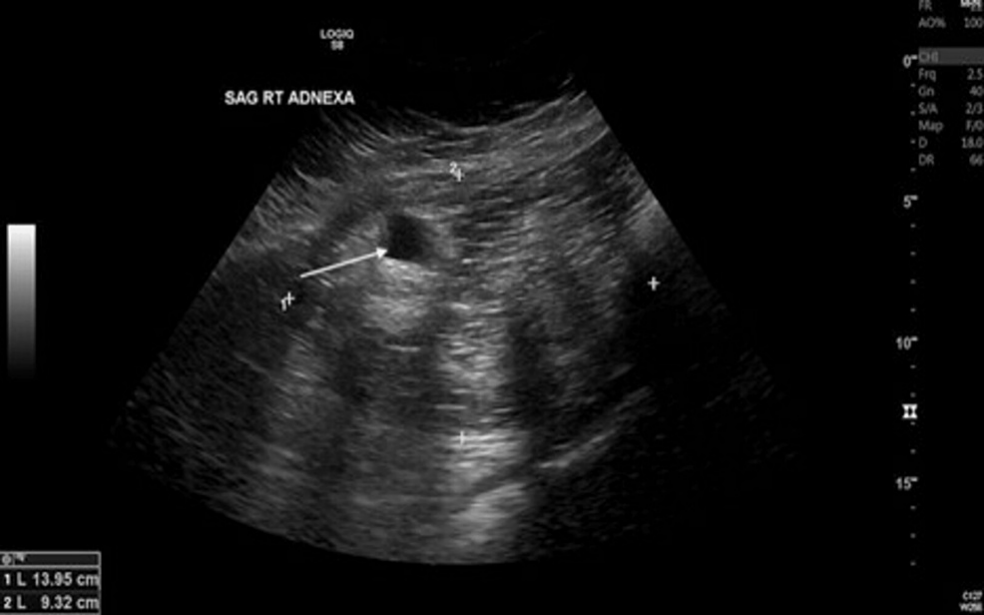
Pelvic ultrasound Arrow denotes ovarian mass.

The pelvic ultrasound showed an endometrial thickening of 2.8 cm (Figure 2). 

**Figure 3:**
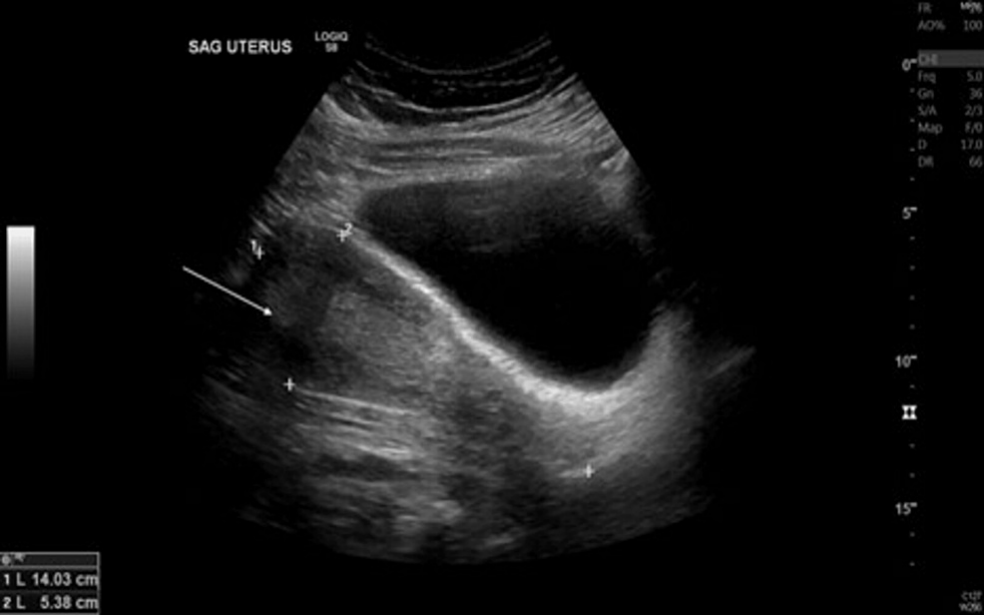
Transvaginal sonogram Arrow denotes ovarian mass; a large dark form is a uterus.

Doppler evaluation on pelvic sonogram did not reveal any evidence of ovarian torsion. A large heterogeneous right adnexal lesion was noted. It was characteristic of a dermoid cyst and comprised of fat and soft tissue (Figure 3). 

The patient was treated with intravenous fluids and given ketorolac for analgesia and ceftriaxone for the urinary tract infection (UTI). The patient was discharged home with a prescription for nitrofurantoin and given follow-up to gynecology for her ovarian mass.

## Discussion

Typically found in the second and third decades of life, dermoid ovarian cysts are the most common germ cell tumor and are difficult to diagnose based on symptoms alone [6]. This may be in part due to their inconspicuous nature. They do not grow quickly and rarely produce symptoms unless secondary to complications [6]. These complications can include torsion, abdominal pain or distension, infection, vaginal bleeding, nausea and vomiting, rupture, and on rare occasions, active hormone production [6,7]. The larger the cyst, the greater the risk of torsion. In two applicable case studies involving ovarian dermoid tumors, prolactin levels were found to be associated with dermoid tumors [6,8]. The high likelihood of an ovarian cyst going undetected makes imaging essential to its diagnosis.

For all asymptomatic women, regardless of whether they are pre- or postmenopausal, a transvaginal ultrasound scan, as was used in this case, is preferred [2]. Dermoid cysts are fairly simple to recognize on pelvic imaging by the presence of bones or teeth [1]. However, in some cases, it is easy to confuse these findings with a complex hemorrhagic cyst. In these situations, signs such as the "dot-dash" sign occur when hyperechoic lines and dots resembling Morse code appear on the image. These "dots" and "dashes" originate from the hairs in the dermoid cyst. Due to their strength in detecting fat, cross-sectional CTs and magnetic resonance imaging (MRI) are very effective for detecting dermoid cysts as well [3].

Several different surgeries are used to remove ovarian dermoid cysts. Operative laparoscopy tends to be preferred to a laparotomy due to benefits such as less blood loss, reduced pain, and a more pleasing cosmetic result; however, it also has a longer operating time and a higher chance of cyst spillage, in addition to a higher recurrence rate [2]. Cyst spillage on rare occasions causes chemical peritonitis and can be challenging to treat. However, if it does occur, it is more easily treated during laparoscopy [2]. In the case of ovarian cystectomy, as opposed to oophorectomy, fertility status is typically used to determine a course of action. Cystectomy is commonly chosen for younger women unless the patient dictates otherwise, and oophorectomy is more common for post and perimenopausal women [2].

Ovarian cancer, behind endometrial and cervical cancer, is the third most common female cancer [9]. Fortunately, dermoid tumors are rarely malignant [1]. Only one to two percent of ovarian dermoid cysts are malignant, and they are more common in women fifty years and older [7]. A dermoid tumor is malignant only when the tissues are immature. However, although a tumor may be primarily benign, there could be some immature, malignant components still present [1]. Cancers, such as squamous cell carcinoma, adenocarcinoma, melanoma, thyroid carcinoma, and many others, can originate from the dermoid cyst [7]. There are also non-ovarian cancers that can mimic ovarian cancer. One such cancer is primary fallopian tube carcinoma. It typically occurs in women 40-65 years old and is a very rare tumor. However, the prevalence estimate of the tumor could be hindered by an incorrect diagnosis. It can present similarly to an ovarian cyst with symptoms such as vaginal bleeding and abdominal pain [10]. Up to seventy percent of tumors are in phase three or four when they are diagnosed [8]. Surgery, radiotherapy, and chemotherapy are typically used to manage it. However, the survival rate is 30% over five years. In situations of early detection, however, it increases to 90% [8]. This underscores the importance of follow-up assessment even if the lesion is asymptomatic and found incidentally on imaging.

## Conclusions

Ovarian dermoid cysts, while mostly benign, do have the potential to cause serious health issues if untreated in the early phases. However, as demonstrated in this case, they can occur even in the absence of positive family history. The earlier a dermoid cyst is found, the lower the morbidity for the patient. While a definitive diagnosis may not be possible in the ED, the liberal use of ultrasonography is helpful in detecting ovarian masses. 

## References

[1] Mahe E, Sur M (2011). Squamous lesions of the ovary. Arch Pathol Lab Med.

[2] Sinha A, Ewies AA (2016). Ovarian mature cystic teratoma: challenges of surgical management. Obstet Gynecol Int.

[3] Sahin H, Abdullazade S, Sanci M (2017). Mature cystic teratoma of the ovary: a cutting edge overview on imaging features. Insights Imaging.

[4] Multani J, Kives S (2015). Dermoid cysts in adolescents. Curr Opin Obstet Gynecol.

[5] Ulbright TM (2005). Germ cell tumors of the gonads: a selective review emphasizing problems in differential diagnosis, newly appreciated, and controversial issues. Mod Pathol.

[6] Elms AF, Carlan SJ, Rich AE, Cerezo L (2012). Ovarian tumor-derived ectopic hyperprolactinemia. Pituitary.

[7] Shiravani Z, Najib FS, Momtahan M, Robati M, Hajisafari Tafti M, Namazi N (2020). Are ovarian dermoid cysts should be always considered benign? A case series study of different malignant transformation. Indian J Surg Oncol.

[8] Klonoff DC, Kahn DG, Rosenzweig W, Wilson CB (1990). Hyperprolactinemia in a patient with a pituitary and an ovarian dermoid tumor: case report. Neurosurgery.

[9] Dong X, Men X, Zhang W, Lei P (2014). Advances in tumor markers of ovarian cancer for early diagnosis. Indian J Cancer.

[10] Shetty M (2019). Nonovarian mimics of ovarian malignancy. Semin Ultrasound CT MR.

